# Silent struggles: Parkinson's disease with multiple system atrophy

**DOI:** 10.51866/mol.612

**Published:** 2024-03-05

**Authors:** Khasnur Abd Malek

**Affiliations:** 1 MBChB, MFamMed, FRACGP, Primary Care Medicine Department, Universiti Teknologi MARA (UiTM), Sungai Buloh, Selangor, Malaysia. Email: drkhasnur@uitm.edu.my

**Keywords:** Multisystem atrophy, Parkinson disease, Neuron degeneration, Autonomic disorders

## Introduction

This poem delves into the insidious nature of Parkinson's disease with Multiple System Atrophy, portraying it as a silent killer that slowly strips individuals of their freedom from within. It sheds on the significant experience observed in a patient who is affected by this complex neurological condition.

**A poem from the perspectives of daughter and a family medicine doctor.**

This poem is written by a grieving daughter who is also a family medicine doctor, in memory of her father. This poem honors a resilient man who faced Multiple System Atrophy with grit, perseverance and courage. My special thanks go to his dedicated wife, the main caregiver, whose love and strength were a guiding light. I thank from the bottom of my heart, the support of his two sons and all family members. A big gratitude to the neighbors, relatives, and helpers for easing his journey. A big thank you to the medical team–specialists, nurses, physiotherapists, speech therapists, and ward staff–for their crucial role in his care and his numerous admissions. Your collective compassion created a lasting tapestry of love and support.

Oh Father,
As multiple system atrophy (MSA) claims its
ruthless call,
Casting its shadows at the evening’s fall
A daughter weeps, her heart in anguish.
Gazing upon a father’s plight

Oh Father,
You endure a rare and relentless fight
As multiple systems weaken, crumble, and fall,
Parkinson’s grip, like a silent veil,
Traps your individual, once strong and proud.

Oh Father,
Lost in shadows, independence shreds
In your own body, a captive embrace.
A once strong man, now confined to his bed,
Trapped in a wheel-chair, bound by despair.

Oh Father,
Each step-down, a silent, mournful toll,
Sleep impaired, anxiety takes control.
Weight diminishing, muscle wasting away,
Muscles stiffening, speech a whispering breeze,

Oh Father,
I watched with heavy heart and fading hope,
Vibrant characters stolen by the grip of disease’s
dismay
Your smile concealed, in an expressionless grace,
Your loving smile now taken away

Oh Father
Independence lost, a vibrant past erased,
Society’s contributor, now tightly encased.
Even as the body succumbs to fate,
Life advice bestowed, a legacy innate.

Oh Father
With a heart heavy, yet somehow light,
I watch your return to your loving Creator,
With grace and serene tranquility
No more the chains of illness to bear

Oh Parkinson
I speaks to you, MSA, with rage unbound,
For the suffering caused, a heartache profound.
You are a torment that won’t ease.
That stole my father a piece at a time.

Oh Parkinson
Oh, ruthless foe, with no compassion in your
trait,
My tears, a torrent, can’t allay.
In my words, a daughter’s plea,
For a world without MSA’s tyranny.

